# Alemtuzumab Plus Cyclosporine Treatment of the Autoimmune Hemolytic Anemia in an Adult Bowel Transplant

**DOI:** 10.1155/2014/262953

**Published:** 2014-08-11

**Authors:** A. Lauro, M. Stanzani, C. Finelli, C. Zanfi, M. C. Morelli, E. Pasqualini, A. Dazzi, M. Ravaioli, M. Di Simone, V. Giudice, L. Pironi, A. D. Pinna

**Affiliations:** ^1^Liver and Multiorgan Transplant Unit, Sant'Orsola-Malpighi University Hospital, 40138 Bologna, Italy; ^2^Institute of Hematology “L. e A. Seràgnoli”, Sant'Orsola-Malpighi University Hospital, 40138 Bologna, Italy; ^3^Immunohematology Service and Blood Bank, Sant'Orsola-Malpighi University Hospital, 40138 Bologna, Italy; ^4^Center for Chronic Intestinal Failure, Sant'Orsola-Malpighi University Hospital, 40138 Bologna, Italy

## Abstract

An adult male underwent a bowel transplant for tufting enteropathy, receiving alemtuzumab, tacrolimus, and steroids as immunosuppressants. Five years later, he developed an autoimmune hemolytic anemia (AIHA), anti-IgG positive, with reduced reticulocyte count, leukopenia, and thrombocytopenia with antiplatelet antibodies. After an unsuccessful initial treatment with high dose steroids, reduction in tacrolimus dose, and intravenous immunoglobulin (IVIG), a bone marrow biopsy revealed absence of erythroid maturation with precursor hyperplasia. The patient was switched to sirolimus and received four doses of rituximab plus two courses of plasmapheresis, which decreased his transfusion requirements. After a febrile episode one month later, the AIHA relapsed with corresponding decreases in platelet and leukocyte count: cyclosporine A (CsA) was started with a second course of rituximab and IVIG without response, even though repeat bone marrow biopsy did not reveal morphology correlated to an acquired pure red cell aplasia (APRCA). Considering the similarity in his clinical and laboratory findings to APRCA, alemtuzumab was added (three doses over a week) with CsA followed by steroids. The patient was eventually discharged transfusion-independent, with increasing hemoglobin (Hb) levels and normal platelet and leukocyte count. One year later he is still disease-free with functioning graft.

## 1. Introduction

Autoimmune cytopenias are a rare but severe complication after hematopoietic stem cell and solid organ transplantation and have been described after pediatric intestinal transplantation [1, 2]. Transplant-associated hemolytic anemia is most commonly reported in nonidentical ABO bone marrow transplants, due to ABO antibodies that cause hemolysis within days to weeks or, when mediated by autoantibodies, months to years after the transplant (autoimmune hemolytic anemia (AIHA)) [3–5]. Most cases of AIHA in solid organ transplant recipients have been reported in the first year after transplant: Botija and coworkers described an AIHA incidence of 12% after pediatric intestinal transplants, most frequently associated with cold agglutinins followed by warm and mixed types [1]. Although tacrolimus has dramatically improved the outcomes of bowel transplantation, persistent T-cell inhibition may favor polyclonal B proliferation and production of autoantibodies [6]. Additional evidence suggests that tacrolimus impairs thymus function, possibly interfering with the process of negative T-cell selection, especially in children. Alternatively, Lacaille et al. did not report signs of dysimmunity in children treated with cyclosporine A (CsA) probably because the drug has a weaker interference on the immune pathways [7]. Only few patients with posttransplant AIHA respond to steroid-based immunosuppression: more frequently, patients require high dose intravenous immunoglobulin (IVIG), rituximab, plasmapheresis, splenectomy (patient's own spleen or transplanted spleen), alemtuzumab, or chemotherapy with cyclophosphamide and vincristine [8]. Herein, we report the case of a young adult, who received a small bowel transplant for tufting enteropathy [9], that developed late-onset AIHA associated to pancytopenia. His anemia, which was IgG positive and associated with reduced reticulocyte count, leukopenia, and thrombocytopenia with intrabone marrow hemolysis, was resistant to three lines of immunosuppressive therapy. He was eventually treated successfully using a therapeutic approach similar to that required for an acquired pure red cell aplasia [10], a syndrome characterized by severe normochromic, normocytic anaemia associated with reticulocytopenia and absence of erythroblasts in the bone marrow.

## 2. Case Report

**Figure 1:**
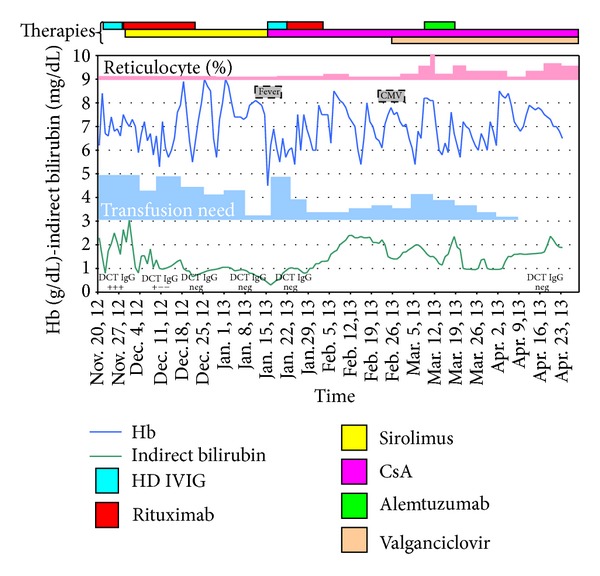
Correlation between therapy and outcome during hospital admission.

A 24-year-old male, blood group O positive, was transplanted on May 2007 for tufting enteropathy with an isolated bowel graft from a O negative female donor, preserving his spleen during the transplant. His preconditioning regimen consisted of two doses of alemtuzumab (0.3 mg/kg) followed by tacrolimus (daily through level 10 ng/mL) and low dose steroids (5 mg prednisone). His posttransplant medical course was complicated by one episode of enteritis, requiring hospital admission on March 2010 and medical management. In November 2012 he developed mild fatigue and jaundice with laboratory evidence of hemolysis. At admission blood tests showed severe normocytic anemia (Hb 6.2 g/dL) with reticulocyte count significantly reduced to 0.4%, thrombocytopenia (16.000/10^9^ L), and 3.000 leukocytes/10^9^ L (neutrophil 90%). The unconjugated (indirect) bilirubin was 2.29 mg/dL, while the other parameters of hemolysis were normal (haptoglobin and lactate dehydrogenase (LDH)). Workup for gastrointestinal bleeding, viral infections (absence of specific anti-parvovirus IgM and IgG antibodies and negative parvovirus B19 PCR in the peripheral blood), or posttransplant lymphoproliferative diseases was negative. He was found to be anti-IgG strongly positive in Direct Antiglobulin Test (DAT) for anti-IgG with antiplatelet antibodies, consistent with a clinical pattern of the Evans syndrome (ES). He required an average of 8–10 red blood cell (RBC) concentrate transfusions per week, maintaining Hb level between 5.3 and 7.9 g/dL. Initially he was treated with high dose steroids (1 gr IV followed by tapering) in combination with high dose IVIG (0.5 mg/kg/day for 6 consecutive days), with reduction in his tacrolimus dose. Granulocyte-colony stimulating factor (G-CSF) was added when leukocyte count was less than 1.500/10^9^ L. Unfortunately, the anemia, leukopenia, and thrombocytopenia were unresponsive to treatment and a bone marrow biopsy revealed absence of erythroid maturation with hyperplasia of the precursors, several megakaryocytes, and granulocytopoiesis with prevalence of immature cells. Considering the resistance to previous treatment, tacrolimus was switched to sirolimus (plus steroids) and a second-line treatment was started with rituximab 375 mg/m^2^ IV per week for 4 consecutive weeks, with the addition of levofloxacin and posaconazole as antibacterial and antifungal prophylaxis. Moreover, he was treated with two courses of plasmapheresis with the intention to clear the autoantibodies. The patient's clinical and laboratory parameters improved on the new regimen, with decreased transfusion requirements (median of 3 transfusions per week to maintain Hb level between 6.5 and 8.7 g/dL) and normalizing platelet count. His leukocyte count was also maintained over 1.500/10^9^ L with a decrease of G-CSF need. Hemolysis tests showed a slight improvement: the indirect bilirubin decreased to 0.9 mg/dL while DAT became negative, but LDH was constantly >280 U/L; haptoglobin was depleted (<5 mg/dL) and reticulocyte count was still very low (0.2%). The bone marrow cytology and histology was unchanged. Twenty days after rituximab administration, the patient developed a febrile episode with negative blood culture, decreasing Hb (ranging from 4.5 to 6.9 g/dL), leukocytes, and platelets. Hemolysis parameters were stable with constant, negative DAT. A repeated bone marrow cytology and histology showed hyperplastic erythropoiesis with absence of erythroid maturation. Although the bone marrow biopsy never showed a typical pattern correlated to an acquired pure red cell aplasia (APRCA), namely, absence of erythroid lineage and normal appearance of granulocytic precursors and megakaryocytes, his clinical and laboratory features appeared to be consistent with the disease. Sirolimus was changed to CsA and high dose IVIG, followed by rituximab 375 mg/m^2^ IV per week for two consecutive weeks. His anemia and transfusion needs improved modestly on the new regimen but exhibited parameters of persistent hemolysis with indirect bilirubin >1.0 mg/dL, LDH 320 mg/U, and depleted haptoglobin. The DAT was still negative and reticulocyte count showed a constant increase until 3.1%. Eighteen days after the second rituximab cycle and continuous administration of CsA and steroids, the patient developed CMV (*Cytomegalovirus*) reactivation detected by PCR and successfully treated with valganciclovir 900 mg daily. Unfortunately, the leukocytes, platelets, and Hb decreased again, increasing the transfusion need with laboratory evidence of hemolysis. Considering the patient's high transfusion requirement, iron-chelation therapy with deferasirox was added. Eventually, in March 2013 a course of alemtuzumab was administered with the following scheme: 3 mg on day 1, 10 mg on day 2, and 30 mg on day 3 followed by a new steroid recycle (1 mg/kg/day IV for 5 consecutive days plus a rapid tapering). The immunosuppressive treatment used in this clinical case is summarized in Table 1. One month after the last course of alemtuzumab and steroids, the patient was discharged transfusion- and G-CSF-independent (Hb constantly > 7.7 g/dL, reticulocytes 5.8%, leukocytes > 3.000/10^9^ L, and platelets > 100.000/10^9^ L), but with persistent laboratory evidence of hemolysis (indirect bilirubin 2.0 mg/dL, LDH 315 mg/U, and haptoglobin < 5 mg/dL). Due to the intensive immunosuppression, the patient was continued on antimould and antiviral prophylaxis with posaconazole and valganciclovir, together with iron-chelating therapy which was switched to deferoxamine (1 gr for 5 days per week subcutaneously) because of kidney intolerance to deferasirox. His maintenance immunosuppression consisted of cyclosporine and low dose steroids. In April 2014, one year after the treatment with alemtuzumab, the recipient has no signs or symptoms related to infection or neoplastic disease and has normal leukocyte and platelet count; he is transfusion-independent (Hb level constantly > 11 g/dL) without evidence of AIHA. His graft is functioning without episodes of rejection or enteritis. The clinical management during hospital admission is summarized in Figure 1.

## 3. Discussion and Conclusions

Multiple therapeutic modalities have been proposed to treat AIHA after solid organ transplant and pediatric patients with refractory AIHA may respond to a switch from CNI-immunosuppressant like tacrolimus to an mTOR-inhibitor [11]. An alternative approach could be the use of alemtuzumab, acting against the pan-lymphocyte antigen CD52 and resulting in adepletion of the T- and B-cell compartment. While B lineages recover rapidly after alemtuzumab administration, T-cells CD8+ and, overall, CD4+ show persistent and profound depletion lasting for months. Willis and colleagues reported that subsequent maintenance with low dose CsA may be required to prevent relapse, especially in nontransplanted populations [12, 13]. In our case, five years after the bowel transplant, an adult patient presented a severe and resistant AIHA due to warm IgG antibodies, associated with thrombocytopenia and leucopenia. His persistent severe anemia responded only to fourth-line immunosuppressive treatment represented by a combination of low dose CsA and alemtuzumab. A splenectomy was not necessary because the autoimmune disorder was also an extravascular disease located inside the bone marrow. Rituximab is considered to be an effective therapy for warm AIHA [2, 7, 8, 11], but relapses such as in our case are possible. We speculated that his anemia was due to not only the AIHA (which responded to rituximab with the disappearance of the IgG autoantibodies in the blood), but also the absence of erythroid maturation in the bone marrow, probably due to a cell-mediated mechanism. Indeed our patient did not benefit from the switch from tacrolimus to sirolimus. Therefore, we managed the patient as if he had APRCA, even if the bone marrow findings were atypical for this anemia, and he required intensive anti-T-cell immunosuppression (CsA plus alemtuzumab) [12]. Our hypothesis is supported by the fact that the bone marrow examination showed not only an impairment of the erythropoiesis, but also a severe reduction of the more mature granulocyte precursors and a clear increase of the megakaryocytes, consistent with impaired myeloid maturation sustained by a cell-mediated mechanism. We cannot rule out an additional benefit of iron-chelation therapy, which has been reported to improve in some cases the bone marrow erythroid function and maturation. The first response was seen one month after alemtuzumab (also 3 months after CsA), and the patient reached a complete remission after three more months. In conclusion, the presence of autoimmune anemias after intestinal transplantation is calling for a deep haematological workup in order to focus the multimodality therapy required to treat the disease. Our case responded to a differentiated multistep immunosuppressive strategy but a further follow-up will be necessary in order to evaluate its effects on long-term outcome.

## Figures and Tables

**Table 1:** Immunosuppressive therapies and response.

	Response to therapy		
	Transfusion need	Indirect bilirubin	DAT
From November 20, 2012, to November 26, 2012	No	No	No
HD PDN + HD Ig			

From November 28, 2012, to January 15, 2013	Yes	Yes	Yes
Rituximab (4) + PA (2) + sirolimus			

From January 16, 2013, to March 06, 2013	No	No	Yes
Rituximab (2) + HD Ig + CsA			

From March 07, 2013, to April 10, 2014	Yes	Yes	Yes
Alemtuzumab (3) + HD PDN + CsA			

HD PDN = high dose prednisolone; HD Ig = high dose immunoglobulin; PA = plasmapheresis; CsA = cyclosporine A; DAT = Direct Coombs Test.

## Abbreviations

AIHA: Autoimmune hemolytic anemia
IVIG: High dose intravenous immunoglobulin
CsA: Cyclosporine A
APRCA: Acquired pure red cell aplasia
Hb: Hemoglobin
DAT: Direct Coombs Test
RBC: Red blood cell
G-CSF: Granulocyte-colony stimulating factor.
